# Improving immunization data quality in Peru and Mexico: Two case studies highlighting challenges and lessons learned

**DOI:** 10.1016/j.vaccine.2018.10.083

**Published:** 2018-11-29

**Authors:** Silas P. Trumbo, Marcela Contreras, Ana Gabriela Félix García, Fabio Alberto Escobar Díaz, Misael Gómez, Verónica Carrión, Karim Jaqueline Pardo Ruiz, Renee Aquije, M. Carolina Danovaro-Holliday, Martha Velandia-González

**Affiliations:** a Vanderbilt University Medical Center, Nashville, TN, United States; b Pan American Health Organization, Comprehensive Family Immunization Unit, Washington, DC, United States; c External Consultant to PAHO, Bogota, Colombia; d Health Secretariat, Mexico City, Mexico; e Ministry of Health, Lima, Peru; f World Health Organization, Expanded Programme on Immunization, Department of Vaccines, Immunizations, and Biologicals, Geneva, Switzerland

**Keywords:** Electronic immunization registries, Immunization information systems, Immunization data, National immunization programs, Global vaccine action plan

## Abstract

**Introduction:**

The Global Vaccine Action Plan and the Regional Immunization Action Plan of the Americas call for countries to improve immunization data quality. Immunization information systems, particularly electronic immunization registries (EIRs), can help to facilitate program management and increase coverage. However, little is known about efforts to develop and implement such systems in low- and middle-income countries. We present the experiences of Mexico and Peru in implementing EIRs.

**Methods:**

We conducted case studies of an EIR in Mexico and of a population registry in Peru. Information was gathered from technical documents, stakeholder focus groups, site visits, and semi-structured interviews of national stakeholders. We organized findings into narratives that emphasized challenges and lessons learned.

**Results:**

Mexico built one of the world’s first EIRs, incorporating novel features such as local-level tracking of patients; however, insufficient resources and poor data registration practices led to the system’s discontinuation. Peru created an information system to improve affiliation to social programs, including the immunization program and quality of demographic data. Mexico’s experience highlights lessons in failed sustainability of an EIR and a laudable effort to reform a country’s information system. Peru’s demonstrates that attempts to improve health and other data may strengthen health systems, including immunization data. Major challenges in information system implementation and sustainability in Peru and Mexico related to funding, clear governance structures, and resistance among health workers.

**Discussion:**

These case studies reinforce the need for countries to ensure adequate funding, plans for sustainability, and health worker capacity-building activities before implementing EIRs. They also suggest new approaches to implementation, including economic incentives for sub-national administrative levels and opportunities to link efforts to improve immunization data with other health and political priorities. More information on best practices is needed to ensure the successful adoption and sustainability of immunization registries in low- and middle-income countries.

## Highlights

• Global and regional action plans call for improved immunization data quality.
• Mexico and Peru created information systems to improve data quality.
• Mexico’s immunization registry failed, but the country is reworking its system.
• Peru’s system has improved data quality, while addressing social priorities.
• Funding and capacity-building affect sustainability of immunization registries.

## 1 Introduction

High-quality immunization data facilitate the management, financial planning, and vaccine forecasting capacities of national immunization programs (NIPs)^1^ [1], [2]. A prerequisite for quality data is an information system that monitors vaccine administration and facilitates the aggregation and analysis of coverage information.

Since 2002, the Pan American Health Organization’s (PAHO) Technical Advisory Group (TAG) on Vaccine-preventable Diseases for the Region of the Americas has issued recommendations for countries to improve the quality of their immunization data and information systems [3], [4], [5], [6], [7], [8]. In addition to implementing plans of action for improving data quality, PAHO has provided technical assistance to countries developing or upgrading electronic immunization registries (EIRs) [6], [9], [10]. In 2009, PAHO’s TAG recommended that countries document their experiences with electronic immunization registries [5]. Additionally, the Global Vaccine Action Plan 2011–2020 (GVAP) and the Regional Immunization Action Plan 2015–2020 (RIAP) in the Americas reinforced the importance of improving immunization data quality [11], [12].

As confidential, computerized, population-based registries that track administered vaccine doses, EIRs serve important functions at all levels [6]. Locally, registries enable health workers to identify persons due for vaccination and to monitor defaulters [13], [14], [15]. Sub-nationally and nationally, EIRs facilitate coverage monitoring by vaccine, dose, age, and other variables [6], [13], [16]. EIRs also facilitate analysis of vaccine safety data, support research initiatives, promote better understanding of vaccine refusals, and inform the design of coverage interventions and outbreak investigations [13], [17].

In the last decade, countries in the Americas have transformed their immunization information systems [16]. As of 2017, 13^2^ countries and territories have introduced EIRs and eight^3^ are creating or implementing these systems [18]. Recent developments include incorporating EIRs into larger health information systems and linking registries to mobile technologies [16], [19]. In 2014, Danovaro-Holliday et al. identified lessons learned on EIR implementation. These included the need for permanent financial and human resources and the value of making EIRs useful for health workers to ensure long-term sustainability of the systems [16].

Despite the importance of strengthening data quality to the GVAP, there are few published reports on efforts to implement EIRs in low- and middle-income countries [10], [20], [21]. In this article, we present the experiences of Mexico and Peru in implementing person-based information systems. We describe how these systems developed as well as the challenges and lessons learned in their implementation. Our findings should be useful to countries seeking to improve immunization data quality and to those developing or strengthening EIRs.

## 2 Methodology

In 2015 and 2016, we conducted case studies of immunization information systems in Mexico and Peru. These countries were chosen based on their distinct experiences and potential value to other nations. We chose Mexico to document the experience of one of the largest and most diverse countries in pioneering one of the first EIRs in Latin America and the Caribbean. Additionally, we wanted to highlight lessons learned from Mexico’s attempts to improve data quality after discovering that its system was not producing accurate coverage levels [22]. We chose Peru to document the county’s unique approach of developing a children population registry not only for the NPI but also for social programs, such as a civil registry. In this respect, we wanted to study how EIRs might be connected to other systems and facilitate inter-ministerial coordination. Both cases were evaluated as part of PAHO’s Improving Data Quality for Immunization (IDQi) project [23].

Used commonly in the social sciences, case studies are qualitative evaluations of individuals, institutions, or events that highlight objective data (e.g., facts and key actions) and provide practical information (e.g., reasons for a program’s success) to audiences in similar situations [24], [25]. By distinguishing a subject from its contexts, case studies offer comprehensive explanations of events or developments [24]. In these case studies, the subjects are Mexico and Peru’s EIRs, while the contexts are the countries’ health systems, economic situations, and political structures.

We gathered information by reviewing national documents on vaccination and information systems, holding focus groups of national stakeholders, conducting semi-structured interviews of individuals, and making visits to sites where immunization data are recorded and analyzed. Initial interviews with stakeholders and a review of documentary evidence helped us to develop interview questions related to the structure and evolution of the immunization information system and to challenges with and lessons learned from implementation. In Peru, we conducted twenty semi-structured interviews and two site visits, one in the regional health network in Cusco (Red de Salud Norte) and another in the municipality of Calca in Cusco. During site visits, we met with immunization officials, municipal authorities, statisticians, managers of information systems, and officials responsible for other health programs (e.g., nutrition). In Mexico, one focus group session was held, with participation of officials from key national agencies, including the NIP, General Directorate of Health Information (DGIS), and National Center for Childhood and Adolescent Health (CENSIA).

We analyzed data by reviewing background documents and transcripts of semi-structured interviews. Information was coded with short phrases, and codes were grouped to identify the main factors affecting information systems in Mexico and Peru [26], [27]. To make results more understandable, we organized major findings into challenges and lessons learned.

## 3 Results

### 3.1 Mexico

#### 3.1.1 Health system and National immunization program

In 1973, Mexico established its NIP, which, in 1991, became the Universal Vaccination Program (PVU)^4^ [28]. At the national level, the Secretary of Health manages the PVU, with additional oversight from the Social Security Institute (IMSS) and the Institute of Social Security and Services for State Workers (ISSSTE). At the local and state levels, health services and the IMSS, ISSSTE, and other public agencies implement vaccination activities and partner with health institutions to offer immunization services. National and state immunization technical advisory groups collaborate with the PVU to standardize schedules and ensure vaccine availability [29].

#### 3.1.2 Immunization information system

In 1991, Mexico established PROVAC, a computerized program to record immunization data in all of the country’s health facilities, including private facilities [28] (Table 1). In 1995, the PVU began organizing immunization records in PROVAC by vaccine type. At this time, the Secretary of Health added nutrition indicators (weight and height), and, in 1999, a unique identifier was added to reduce the number of duplicate entries. In 2000, officials separated administrative records from national census data and started using only PROVAC to determine numerators to calculate coverage rates, using population projections as denominators.

**Table 1:** Description of PROVAC, immunization information system in Mexico.

Description: Established in 1991, PROVAC served as the electronic immunization registry of Mexico until 2013. Though not online, PROVAC was a computer-based system that registered the immunization status of children and pregnant women. The registry included each federal entity’s data and was maintained until 2013					
Objectives	Information included	Flow of information	Government entities involved	Technical support	Legal basis
1. Estimate vaccination coverages 2. Facilitate follow-up of vaccination schedules of children aged <8 years	Demographics: – Name – Birthdate – Sex – Names of parents or guardians – Address – National identification documents (NIDs) Health data: – Vaccination site – Dose, date, and antigen of vaccine administered – Nutritional data – Reasons for lack of vaccination	– Health workers enter children’s data in a computer-based system – Denominators (i.e., target populations) based on local censuses and the National Population Council (CONAPO) – Numerators based on consolidated records of administered doses	– Secretary of Health – CONAPO	– Secretary of Health through National Center for the Health of Children and Adolescents (CENSIA)	– *Official Mexican Policy NOM-004-SSA3-2012* on the medical chart – No clear legal basis for the system

Though official coverage rates were high during this time, there were concerns that the levels might be falsely elevated. During the system’s 18-year history, Mexico developed different versions of PROVAC both because the program was open-source and because changes were required to include eight vaccines added to the PVU from 1991 to 2008.

In 2013, Mexico stopped using PROVAC due to coverage discrepancies. The National Population Council (CONAPO) had completed a data reconciliation with information from the National Institute of Statistics and Geography, which showed that PROVAC’s denominators were underestimated and that coverages were thus overestimated. Coverages estimated at near 100% dropped to around 80% (e.g., 99% DTP3 for 2012 vs. 83% DTP3 for 2013) [30], [31].

#### 3.1.3 Implementation challenges

PROVAC faced many challenges, including the accelerated inclusion of new vaccines and insufficient resources and information technology personnel devoted to the system (Table 2). Furthermore, interviewed officials at different levels of the Mexican health system indicated that the major cause of PROVAC’s discontinuation was poor data recording practices.

**Table 2:** Challenges and lessons learned, PROVAC, immunization information system in Mexico.

*Challenges*	
Governance and sustainability	*Lack of central funding for staffing and system maintenance
Human resources	*Difficulty securing and retaining information technology staff
Processes	*No evaluation comparing the system’s estimates to those from other data sources, eventually resulting in a large coverage adjustment and more work in the long-term *Insufficient ability to adapt to different users' information needs and to changes in national circumstances
Tools	*Open-source and open-access program resulting in multiple versions of the same program, in which different institutions modified the software according to their preferences
Data use and data quality	*Lack of a culture supporting high quality data practices at all levels

*Lessons learned*	
Governance and sustainability	*Activities empowering community members can promote program sustainability *Value of involving the private sector in all stages of implementation *Empowerment and involvement of local governments *An eHealth strategy allows for a sustainable framework for developing the country's health system
Human resources	*Promote efficient staffing *Upon implementing a new system, as done after PROVAC, the country should expect resistance on the part medical units responsible for data registration. One solution is to carry out progressive trainings and educational activities in these units.
Processes	*Improved ability to quantify target population
Tools	*Use of single information system may result in time-savings
Data use and data quality	*In calculating coverages, the sources of the numerators and denominators must be determined to anticipate possible causes of underestimates or overestimates. Mexican officials did not realize that the numerator in the PROVAC system was based on doses distributed doses, rather than administered doses. This resulted in overestimated coverage. *Complete systematic evaluations of data quality as part of the routine program

PROVAC’s open coding exposed it to errors. Between and within regions, users and administrators created different versions of the system, resulting in inconsistencies and the potential for data manipulation. Complicating matters were errors in numerators and denominators. Denominators were never validated against CONAPO’s data, and some numerators had been based on distributed rather than administered doses. This resulted partly from PROVAC’s design. Although health workers linked administered doses to patients at the local level, the system was never online, meaning coverage information at higher levels was based on aggregated data and prone to substitute estimations (i.e., doses distributed rather than doses administered).

#### 3.1.4 Lessons learned and next steps

In 2013, Mexico acknowledged the poor quality of vaccination coverage estimates and began to revamp its information system (Table 2). While developing a new EIR, the country returned to using administrative doses to calculate coverage, started to modernize its informatics system, and began to revise local and regional population estimates. These efforts have been globally recognized, and other countries have been encouraged to follow Mexico’s example of transparency and accountability [31].

Nevertheless, the transition has been difficult due to challenges in coordinating public and private providers of immunization services and because of Mexico’s fragmented health system with multiple providers and health insurance mechanisms [32], [33]. Some health workers resent returning to recording administered doses on paper, and since the system is no longer individualized, it may be more difficult to identify undervaccinated children. To address these challenges, the government has held trainings for health workers on why changes to the EIR occurred and on how to use the new system.

### 3.2 Peru

#### 3.2.1 Health system and the National immunization program

Peru established its NIP in 1979. Coverages have generally been high (DTP3 88–95% between 2012 and 2016) [34]. In the early 2000s, Peru began decentralizing its government, and the NIP was integrated into the Ministry of Health’s (MoH) Comprehensive Child Health Care program [35], [36]. Renamed the National Immunization Strategy (ESNI) in 2004, the immunization program offers 15 free vaccines [35]. National agencies involved in immunization include the Ministry of Economics and Finance (MEF); the Ministry of Development and Social Inclusion (MIDIS), which oversees efforts to reduce health inequalities; and the National Registry of Identification and Civil Status (RENIEC), which is charged with issuing each Peruvian a National Identification Document (NID) [37], [38].

#### 3.2.2 The Padrón Nominal and its impact on immunization data

In 2012, Peru created a census database, the *Padrón Nominal* (PN), of children aged <6 years to improve affiliation to social programs and the quality of health and demographic data (Table 3).

**Table 3:** Description of the Padrón Nominal, immunization information system in Peru.

Description: Established in 2012, the Padrón Nominal is a census database that allows online registration of children aged <6 years. The system operates in approximately 2500 health facilities. Health workers use the system to verify children’s identities and to update and validate their immunization records					
Objectives	Information included	Flow of information	Government entities involved	Technical support	Legal basis
1. Provide State programs data for planning and budgeting 2. Identify gaps in insurance and access to health and education services 3. Provide an updated, standardized registry of children aged <6 years at the district level 4. Identify children without national identification documents (NIDs), so that they can be included in civil registries 5. Outfit regional governments with a tool to manage interventions to improve health of children aged <6 years	36 variables in 5 categories: 1. Service site (e.g., health center code) 2. Identification (e.g., birthdate and NID) 3. Participation in educational and social programs (e.g., school code) 4. Relation to head of household (e.g., father’s last name) 5. Mother’s identification and poverty level (e.g., mother’s native language)	-Municipal officials enter children’s data in online system -Denominator (i.e., children aged <6 years) updated via birth certificates issued in hospital and through civil registries and review of NIDs. Municipal and civil registry officials search for new children to include in the system -System updated on daily basis	-Ministry of Health -Ministry of Economics and Finance -National Registry of Identification and Civil Status (RENIEC) -Ministry of Development and Social Inclusion	-National Registry of Identification and Civil Status (RENIEC) is charged with administering the system, including granting access to users and troubleshooting problems -Ministries of Education and Health review processes and technical information as needed	-*Law 29,332 (2013)*: creates “Incentives Plan to Improve Municipal Management” *Supreme Decree 002*–*2013-EF*: Approves procedures to achieve goals and assign resources for “Incentives Plan to Improve Municipal Management” *Resolution 389 (2017)*: Approves the Padrón Nominal at the district level for children aged <6 years

Three major political issues led to the PN’s creation. The first was the longstanding problem of citizens lacking representation in civil registries [38], [39]. In 2010, an estimated 4.7 million Peruvians (16% of the population), 4.6 million of whom were aged <18 years, lacked NIDs, and could not fully access social services or exercise their constitutional rights [39]. Second, growing pressure to reduce economic and health disparities underscored the need to develop systems to detect low vaccination coverages [40]. Finally, the MoH and MEF argued that a database that permitted monitoring of individuals would improve the performance of ESNI and health programs.

Developing the PN required Peru to evaluate its birth registration practices. Since 2004, the ESNI had used a paper-based registry linked to the MoH’s health and target data from census projections and, starting in 2016, had used birth registration systems to calculate immunization coverage [36]. Previously, the MoH required health workers in delivery rooms to record all live births in a computer database [41]. Although this practice should have resulted in the creation of birth certificates and subsequent generation of NIDs, the system missed many children [39].

To capture children without documentation, the MoH, MEF, and MIDIS incentivized municipalities to identify all children aged <6 years in their areas through collaboration with health facilities and civil registration offices. The MEF required local governments to capture all users of immunization services in registries as a condition of financing the PN [42]. Similarly, MIDIS incentivized municipalities to implement the PN through its “Sello Muncipal” program, which provided recognition and financial incentives for municipalities for completing various health indicators, including implementation of the PN. In response, local governments employed different strategies for data collection. Some conducted house-to-house searches; others, particularly large municipalities, requested that health facilities generate lists of all persons aged <6 years in their jurisdictions.

From 2013 to 2016, these incentives led to widespread use of the *Padrón.* The PN started collecting immunization data and soon expanded to nutrition and other health programs. According to local and national officials, the system’s advantages include saved time through the use of a single data entry point for different interventions, better information for planning and decision-making (e.g., more accurate vaccine forecasting), generation of NIDs for children not recorded in the civil registry, and improved identification of target populations for various health interventions. In 2016, MEF decided to stop funding incentives to use the PN, believing that the system had been sufficiently established in the municipalities. However, in 2017, funding resumed, and many municipalities continue to use the system today.

#### 3.2.3 Implementation challenges

**Fig. 1:**
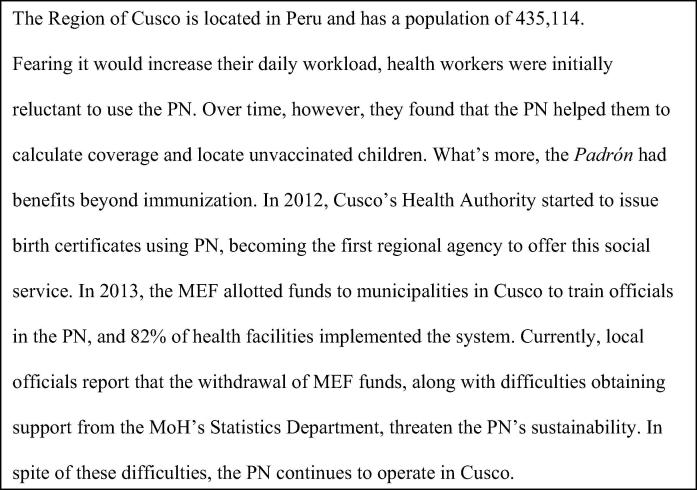
Regional case study, Cusco, Peru.

Challenges in implementing the PN include reconciling discrepancies in denominator data (PN’s population figures vs. census data), difficulties connecting the system to nominal registries, and lack of funding from the MEF in 2016 (Table 4). The Region of Cusco illustrates the PN’s achievements and challenges (Fig. 1).

**Table 4:** Challenges and lessons learned, Padrón Nominal, immunization information system in Peru.

*Challenges*	
Governance and sustainability	*Lack of economic incentives for implementation in 2016 *Change of regional and local governments *Lack of involvement of the private sector *Occasional lack of support from mayors
Human resources	*Health worker perception that more time was spent using the system than serving patients *High labor burden, resulting in incorrect data registration practices *Low health worker payments and high turnover
Processes	*Need to more effectively communicate the PN’s advantages to users, national authorities, and the community
Tools	*Existence of different formats *Lack of connection between the PN with other information systems of the different government entities involved--specifically, the ESNI did not have the PN connected to its immunization information system
Data use and data quality	*Insufficient use of information from the PN by some users and involved programs

*Lessons learned*	
Governance and sustainability	*Empowerment and involvement of local governments *Strengthened cooperation among government agencies in different sectors *Economic incentives serve as good motivation for implementation, but if they are unavailable, municipalities may not continue using the system *Activities empowering community members can promote program sustainability *Value of involving the private sector in all stages of implementation *Coordination between government workers at different levels and in different sectors (i.e., both health and municipal public employees)
Human resources	*Education and training sessions are necessary to counter expected health worker resistance
Processes	*The system facilitates more realistic budgeting practices *Continual feedback on the implementation of the PN was key, and different indicators for monitoring should be established
Tools	*Feasible to link population-based immunization information systems with the nominal registry of Peru
Data use and data quality	*There is multisectoral interest in programs like PN, since their data are useful in different sectors

#### 3.2.4 Lessons learned and next steps

Due to the aforementioned challenges, the PN remains incompletely implemented. Even so, the system’s capacity to identify children lacking health or social interventions and to generate useful data for decision-making suggests that it may become a permanent policy tool (Table 4). The PN has promoted better cooperation among government agencies—MoH (vaccines), MEF (public financing), MIDIS (social programs), and RENIEC (civil registration)—and is an excellent example of a multi-purpose information system. Other lessons learned in implementation include the value of economic incentives, the effectiveness of linking immunization advocacy to larger political movements (e.g., lack of civil registration), and the importance of trainings for health workers and community members. As officials in Cusco indicated, the sustainability of the PN ultimately requires the community’s investment. By educating the public on the benefits of the *Padrón*, including access to health services, civil registration, and social services, parents may come to request the system and increase pressure on the government to sustain the program.

## 4 Discussion

Extending the benefits of immunization to all people requires reliable and timely immunization data [1]. Mexico and Peru are two examples of national initiatives to improve data quality. Having created one of the world’s first EIRs in 1991, Mexico ultimately devoted insufficient resources and attention to PROVAC, resulting in poor data registration practices and the system’s discontinuation. In 2012, Peru created the *Padrón Nominal,* which incorporated some of PROVAC’s best features, including non-vaccine indicators and local-level tracking of patients. Mexico’s experience shows both failed implementation and a laudable effort to reform its data system. Peru’s demonstrates that efforts to improve immunization data may strengthen health systems, increase the uptake of other health interventions, and address political and social priorities.

Compared to other aspects of NIPs in low- and middle-income countries, EIRs may be understudied and underappreciated. While sustainable funding and health worker training are key determinants of EIR sustainability, relatively little is known about how low- and middle-income countries can sustain the systems [14]. Countries likely recognize the importance of key components of an enabling environment—i.e., funding, capacity building, and a trained workforce—but struggle with implementation due to limited resources and competing priorities. Based on Mexico and Peru’s experiences, we wish to highlight four lessons relevant to sustainable implementation of EIRs.

First, rationales for establishing or strengthening EIRs may extend beyond immunization to other health and political priorities. As immunization integrates into other health services, governments are more likely to support proposals for multi-purpose interventions. The PN’s creation in Peru, supported by at least four governmental agencies, exemplifies this point. Second, EIR sustainability requires the investment of various government agencies, and investment might be increased if each agency understands what it stands to gain. In Peru, the MEF sought better data for planning purposes, the MoH sought higher coverages, and the MIDIS sought more equitable health outcomes. In designing EIR proposals and sustainability plans, health officials should thus consider how the system will benefit all stakeholders. Third, predictable funding is necessary, and countries lacking funding should likely delay implementation until resources are available and ideally protected by long-term strategies, with clear governance structures and budgets secured by legislation. Countries may consider innovative funding models, such as the economic incentives used in Peru. These models may facilitate implementation and prove cost-effective if they improve service quality.

Finally, EIR sustainability depends on the system’s quality and adaptability. Recommendations exist for variables, such as the child’s age, and for guiding principles, such as entering data as close to vaccination as possible in terms of time and place [16]. To these, we might add that program developers and national officials must balance pressures to modify the EIR for user preference with the need to maintain a uniform system across the country, particularly as Mexico’s system’s open-source coding contributed to its ultimate downfall. Countries should have information technology personnel devoted solely to the management and updating of the EIR.

Several limitations should be acknowledged. Case studies offer in-depth explanations of complex events with multiple causes but are criticized as lacking rigor and producing non-generalizable results [24]. Although we tried to use thorough qualitative methods, different investigators may have reached different conclusions. In evaluating the applicability of our findings to immunization data quality improvement efforts in other countries, national officials should consider their countries’ health, political, and social factors. Additionally, the PN, though promising, is an ongoing project whose ultimate effectiveness is unknown. Finally, while there is considerable evidence associating EIRs and eHealth initiatives with improved immunization coverage levels, these impact studies are from developed countries [13], [43], [44]. Impact studies of EIRs in low- and middle-income countries are needed to better understand these interventions.

To deliver the benefits of vaccination to all persons, countries in the Americas and other WHO regions must continue to improve their immunization information systems. Mexico and Peru’s experiences should be helpful to other countries seeking to use EIRs. More information on best practices and lessons learned is needed to ensure the successful implementation of EIRs in low- and middle-income countries.

## Declaration of competing interests

The authors have no competing interest to declare.

## Funding

Grant # OPP1084485 (2013), Bill and Melinda Gates Foundation.

## Footnotes

1 National immunization programs (NIPs); Pan American Health Organization (PAHO); Technical Advisory Group (TAG); electronic immunization registries (EIRs); Global Vaccine Action Plan (GVAP); the Regional Immunization Action Plan (RIAP); Improving Data Quality for Immunization (IDQi); General Directorate of Health Information (DGIS), and National Center for Childhood and Adolescent Health (CENSIA); Universal Vaccination Program (PVU); Social Security Institute of Mexico (IMSS); Institute of Social Security and Services for State Workers of Mexico (ISSSTE); National Population Council of Mexico (CONAPO); Ministry of Health (MoH); Ministry of Economics and Finance of Peru (MEF); Ministry of Development and Social Inclusion of Peru (MIDIS); National Registry of Identification and Civil Status of Peru (RENIEC); National Identification Document (NID); and *Padrón Nominal* (PN).

2 Argentina, Belize, Brazil, Chile, Colombia, Costa Rica, Grenada, Guatemala, Honduras, Panama, Uruguay, almost every state in the United States, and some Canadian provinces.

3 Dominican Republic, Ecuador, Peru, Venezuela, and some Caribbean islands (Turks and Caicos, Montserrat, Saint Kitts and Nevis, and Saint Vincent and the Grenadines).

4 All acronyms of public agencies in Mexico and Peru are provided in Spanish (e.g., PVU for *Programa Universal de Vacunación*).
